# Impact of Methyl-β-Cyclodextrin and Apolipoprotein A-I on The Expression of ATP-Binding Cassette Transporter A1 and Cholesterol Depletion in C57BL/6 Mice Astrocytes

**DOI:** 10.22074/cellj.2021.7061

**Published:** 2021-03-01

**Authors:** Shirin Azizidoost, Hossein Babaahmadi-Rezaei, Zahra Nazeri, Maryam Cheraghzadeh, Alireza Kheirollah

**Affiliations:** Cellular and Molecular Research Center, Department of Biochemistry, Medical School, Ahvaz Jundishapur University of Medical Sciences, Ahvaz, Iran

**Keywords:** ATP Binding Cassette Transporter 1, Apolipoprotein A-I, Astrocytes, Beta-cyclodextrin, 3-hydroxy-3-methyl- glutaryl coenzyme A reductase

## Abstract

**Objective:**

Dysregulation of cholesterol metabolism in the brain is responsible for many lipid storage disorders, including Niemann-Pick disease type C (NPC). Here, we have investigated whether cyclodextrin (CD) and apolipoprotein A-I (apoA-I) induce the same signal to inhibit cell cholesterol accumulation by focusing on the main proteins involved in cholesterol homeostasis in response to CD and apoA-I treatment.

**Materials and Methods:**

In this experimental study, astrocytes were treated with apoA-I or CD and then lysed in RIPA buffer. We used Western blot to detect protein levels of 3-hydroxy-3-methyl-glutaryl coenzyme A reductase (HMGCR) and ATP-binding cassette transporter A1 (ABCA1). Cell cholesterol content and cholesterol release in the medium were also measured.

**Results:**

ApoA-I induced a significant increase in ABCA1 and a mild increase in HMGCR protein level, whereas CD caused a significant increase in HMGCR with a significant decrease in ABCA1. Both apoA-I and CD increased cholesterol release in the medium. A mild, but not significant increase, in cell cholesterol content was seen by apoA-I; however, a significant increase in cell cholesterol was detected when the astrocytes were treated with CD.

**Conclusion:**

CD, like apoA-I, depletes cellular cholesterol. This depletion occurs in a different way from apoA-I that is through cholesterol efflux. Depletion of cell cholesterol with CDs led to reduced protein levels of ABCA1 along with increased HMGCR and accumulation of cell cholesterol. This suggested that CDs, unlike apoA-I, could impair the balance between cholesterol synthesis and release, and interfere with cellular function that depends on ABCA1.

## Introduction

Beta-cyclodextrin (β-CD) is reported to be effective in exit of cholesterol from the plasma membrane (1, 2); however, relatively few studies have investigated its mechanism of action in influencing either *in vivo* or *in vitro* cholesterol metabolism, especially in diseases such as Niemann-Pick disease type C (NPC). A number of candidate proteins involved in cholesterol synthesis/ trafficking and efflux have been introduced. In this research, we focused on two proteins of this type, ATPbinding cassette subfamily A member 1 (ABCA1) as the main protein for cholesterol efflux and 3-hydroxy3-methyl-glutaryl coenzyme A reductase (HMGCR) as an important and rate limiting enzyme in cholesterol synthesis (3). 

There is increasing evidence that deregulation of lipoprotein and/or lipid metabolism is coupled to the progression of neurodegenerative diseases like Alzheimer’s disease (AD) and NPC (4, 5). Cholesterol is a primary lipid that regulates brain cell structure and function during the developmental period and adult life (4). The blood brain barrier (BBB) separates the brain´s cholesterol metabolism from the periphery (6); therefore, maintaining the steady-state content of cholesterol in the brain is of particular importance for its physiological function (4). HMGCR acts as a rate-limiting enzyme in cholesterol synthesis and is the primary site of feedback regulation in the biosynthesis of cholesterol (7). ABCA1, a member of the ATP-binding cassette transporters family, is responsible for the majority of cholesterol efflux to deliver cholesterol to an acceptor like apolipoprotein A-I (apoA-I) for high-density lipoprotein (HDL) generation (8). There is abundant evidence that ABCA1-mediated cholesterol efflux to apoA-I can occur at the plasma membrane (9). Thus, the mentioned enzymes are targets of the highly successful blood cholesterol-lowering drugs and their inhibition is a rapid mechanism for switching off the cholesterol synthesis.

Altered brain lipid metabolism, such as cholesterol, has been implicated in the progression of neurodegenerative diseases like NPC and AD (10). Cholesterol reduction in experimental animal models delays the progression of Alzheimer’s pathology. These findings raise the possibility that treating humans with cholesterol lowering medications might reduce the risk of developing AD (11). In other words, it has been reported that the loss of cholesterol shuttling in NPC disease is associated with reduced activity of ABCA1, which is responsible for low HDL cholesterol levels in NPC patients (12).

ApoA-I, a natural cholesterol lowering agent, is one of the main apolipoproteins in the brain. It is an HDL cholesterol transporter that prevents brain cholesterol deposition and holds neuroprotective properties. Decreased serum HDL cholesterol and apoA-I concentration is shown to be highly correlated with AD severity (13). In the human brain, an association has been found between apoA-I with amyloid beta deposits; complexes between apoA-I and amyloid beta can be detected in cerebrospinal fluid (CSF) from AD patients (14).

Cyclodextrins (CDs), namely synthetic cholesterol lowering agents, are a family of cyclic polysaccharide compounds widely used to bind cholesterol. The use of CDs, in particular β-CDs, is increasing in biomedical research because they are able to interact with cell membranes and are known to extract cholesterol and other lipids from these membranes (15). β-CD is a biologically active molecule, and studies have shown that β-CD and its derivatives significantly reduce intracellular cholesterol levels in NPC mutants (16). CDs may also be useful for AD because of intriguing parallels between NPC1 and AD, including neurofibrillary tangles and prominent lysosome system dysfunction (17). 

β-CD has been reported to play a role in cholesterol exit from the plasma membrane (1) but relatively few studies have dealt with its mechanism of action to influence *in vivo* or *in vitro* cholesterol metabolism, especially in certain diseases such as NPC (18, 19). There are a number of candidate proteins implicated in cholesterol synthesis/ trafficking and efflux. Here we focused on two of them: ABCA1, as the main protein of cholesterol efflux, and HMGCR as an essential rate-limiting enzyme in cholesterol synthesis. In the present study, we used a cell culture model to elucidate and compare the mechanism of CD-mediated cholesterol depletion with apoA-I mediated cholesterol efflux from astrocytes through investigating the protein expressions of ABCA1 and HMGCR. 

## Materials and Methods

### Materials

Beta-cyclodextrin (C4805) and a cholesterol quantitation kit (MAK043-1KT) were purchased from Sigma-Aldrich (USA). Dulbecco’s Modified Eagle’s Medium (DMEM; low glucose) and 0.25% trypsin-EDTA were obtained from Bio-Idea (Iran). Mouse anti-ABCA1 monoclonal antibody (cat. no. HJ1) was obtained from Invitrogen (USA), and rabbit anti-HMGCR monoclonal antibody (cat. no.174830) and rabbit anti-GAPDH antibody (Cat. no. 181603) were purchased from Abcam (USA). ApoA-I was a generous gift from Dr. JI. Ito (Biochemistry Dept., Nagoya City University Graduate School of Medical Sciences, Nagoya, Japan). Fetal bovine serum bovine serum (FBS) and penicillin/streptomycin were purchased from Gibco (USA). Hexane and isopropanol were obtained from Merck (Germany).

### Primary isolation and culture of astrocytes

In this experimental study, 18 mice were housed in a temperature-controlled room (24 ± 1˚C) under 12 hours light/dark conditions with free access to food and water. The mice were fed with a standard commercial chow diet and water for a week to stabilize their metabolic condition. The animal procedures were in accordance with the guidelines for animal care prepared by the Committee on Care and Use of Laboratory Animal Resources, National Research Council (USA), and approved by the Institute of Animal Ethics Committee (IAEC) in Ahvaz Jundishapur University of Medical Sciences (AJUMS) for the Purpose of Control and Supervision of Experiments on Animals (IR.AJUMS.REC.1395.637). Astrocytes were isolated from P0 C57BL/6J wild-type mice based on a previously described protocol (20). Briefly, after brain dissection and removal of the meninges, the minced brain pieces were incubated with 0.1% trypsin solution in Dulbecco´s phosphate-buffered saline (DPBS) for 3 minutes at 37˚C to obtain single cells. The cell suspension was centrifuged at 1000 rpm for 1 minute and the cell pellet was cultured in DMEM, low glucose + 10% FBS + 1% penicillin/ streptomycin for one week for the primary culture and a subsequent week for the secondary culture (21). 

### Experimental design and treatment

Astrocytes were plated at a density of 3×10^6^ in DMEM/10% FBS medium, incubated at 37˚C and 5% CO_2_ , and allowed to adhere. Astrocytes that were 75% confluent were treated with 5 µg/ml apoA-I or 5 µM betacyclodextrin for 24 hours. Vehicle-treated cells were used as the control dish.

### Immunoblotting

An equal amount of proteins (150 µg protein/lane) in the cell lysate were separated by 10% sodium dodecyl sulphate-polyacrylamide gel electrophoresis (SDSPAGE) and then transferred to a polyvinylidene difluoride membrane. Bands of HMGCR and ABCA1 were detected after overnight immunostaining of the membrane with specific primary antibodies against HMGCR (1:5000 dilution, Abcam) and ABCA1 (1:2000 dilution, Invitrogen), followed by a subsequent incubation for 2 hours with the corresponding HRP-conjugated anti-IgG (1:4000 dilution, Sigma) as secondary antibodies. Rabbit anti-GAPDH (1:4000 dilution, Abcam) was used as an internal control for equal loading, and immunoreactive proteins were quantified with enhanced chemiluminescence (ECL) reagent followed by densitometric analysis with ImageJ software.

### Extraction of lipid from astrocytes

To determine the cellular cholesterol content, the culture medium was removed and the cells were washed with DPBS. Next, the cell plates were dried with a dryer. We added 1.5 ml of hexane: isopropanol (3:2) solution to each culture plate to extract lipids by shaking the samples for 1.5 hours at room temperature. Then, the supernatant was transferred to a tube and this step was repeated with the same volume of hexane: isopropanol (3:2) for another hour. After evaporating the organic solvent in a 40˚C water bath under nitrogen gas, the dried lipids were dissolved in 200 µl cholesterol assay buffer and vortexed until the mixture was homogenized and stored at -20˚C for further cholesterol assay. 

### Cholesterol assay in cell and conditioned media 

We determined the cholesterol content of the astrocytes and conditioned media based on the protocol presented in the Sigma cholesterol quantitation kit (MAK043-1KT). Briefly, a set of cholesterol standards were prepared by diluting 2 µg/µl stock solution of standard cholesterol provided with the kit. Reaction mixtures were set up according to the kit’s protocol and the absorbance of samples was measured at 570 nm. All samples and standards were run in triplicate and the cholesterol content of the samples was determined from a standard curve. 

### Statistical analysis

Statistical analysis of this experimental study was performed with SPSS (version 18) software. Descriptive statistics presented data as mean ± SD and analysis of variance (ANOVA) was used to check significant differences between groups in the results from Western blotting analysis. In all triplicate experiments, significant differences were noted at * P≤0.05 and **P≤0.01.

## Results

### Characterization of astrocytes

In the previous study, astrocytes isolated by the same method were characterized immunohistochemically with specific anti-glial fibrillary acidic protein (GFAP) antibody. The results showed that the cellular population contained 95% GFAP-positive cells, which are a marker for astrocyte characterization (20, 21). No morphology changes were detected before and after treatment (Fig. S1). (See Supplementary Online Information at www. celljournal.org).

### Effects of apolipoprotein A-I and beta-cyclodextrin on protein levels of 3-hydroxy-3-methyl-glutaryl coenzyme A reductase 

**Fig.1:**
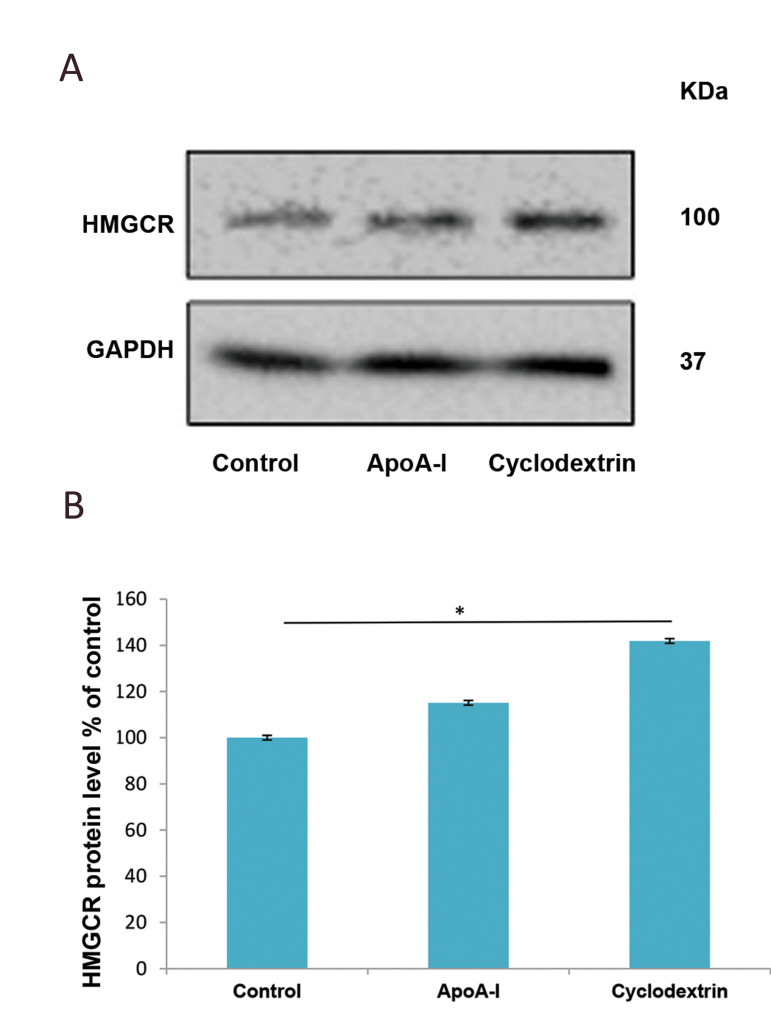
Effects of apoA-I and β-CD on HMGCR protein levels in a primary culture of astrocytes. Mouse astrocytes were incubated with 5 μg/ ml of apoA-I and 5 μM of β-CD. After 24 hours of incubation, the cells were harvested with RIPA buffer. **A.** Then, 150 μg/lane of cell lysate was subjected to SDS-PAGE and western blot analysis against the HMGCR antibody. **B.** The bands were scanned and normalized with β-actin as an internal control. Data were analysed with SPSS and represent mean ± SD of triplicate samples. *P<0.05 indicates statistical significance. apoA-I; Apolipoprotein A-I, β-CD; Beta-cyclodextrin, HMGCR; 3-hydroxy3-methylglutaryl coenzyme A reductase, and SDS-PAGE; Sodium dodecyl sulphatepolyacrylamide gel electrophoresis.

In order to check the effect of apoA-I and β-CD on the protein level of HMGCR, which is the main ratelimiting enzyme involved in cholesterol synthesis, we treated the cultured astrocytes with 5 µg/ml of apoA-I or 5 µM of β-CD for 24 hours. Once the cells were harvested, cell lysates were subjected to SDSPAGE and HMGCR was detected by western blot. As indicated in Figure 1, both apoA-I and β-CD increased the protein level of HMGCR, which was only significant for β-CD treatment with a 51% increase in comparison to the control group (Fig .1). 

### Effect of apolipoprotein A-I and beta-cyclodextrin on protein levels of ATP-binding cassette transporter A1

**Fig.2:**
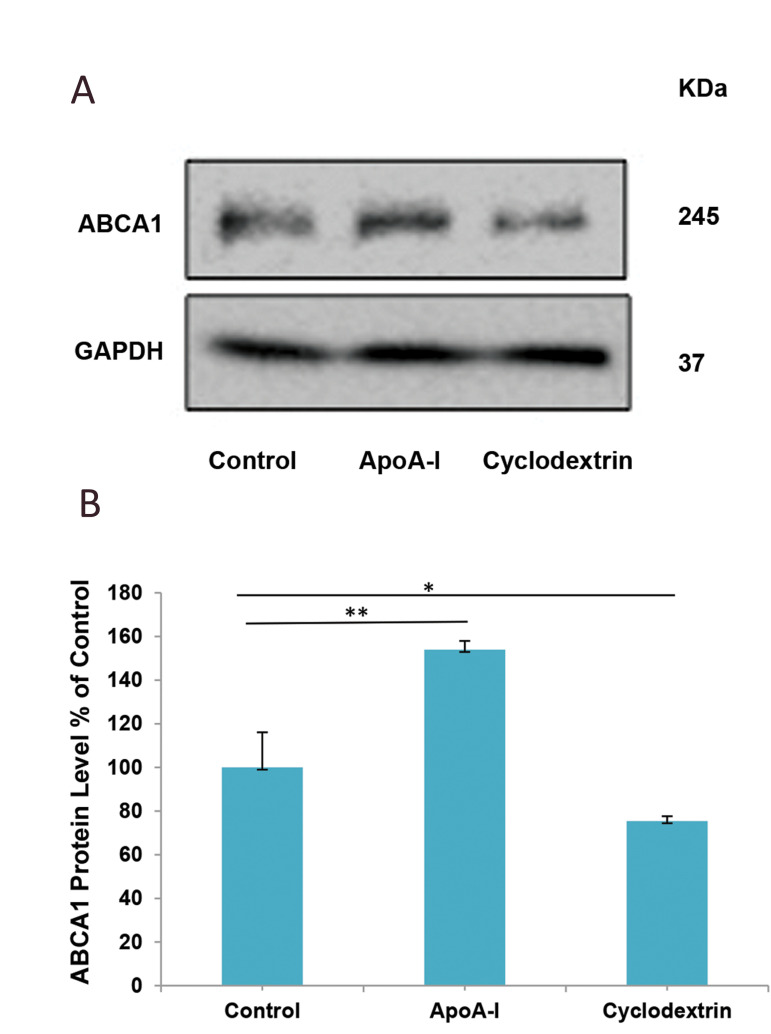
Effect of apoA-I and β-CD on protein level of ABCA1 in primary culture of astrocytes. Mouse astrocytes were incubated with 5 μg/ml of apoA-I and 5 μM of β-CD. After 24 hours of incubation, the cells were harvested with RIPA buffer and **A.** 150 μg/lane of cell lysate was subjected to SDS-PAGE and Western blot analysis against the ABCA1 antibody. **B.** The bands were scanned and normalized with β-actin as an internal control. Data were analysed with SPSS and represent mean ± SD of the triplicate samples. *P<0.05 indicates statistical significance. apoA-I; Apolipoprotein A-I, β-CD; Beta-cyclodextrin, ABCA1; ATP-binding cassette transporter A1, SDS-PAGE; Sodium dodecyl sulphate-polyacrylamide gel electrophoresis.

We sought to investigate the effects of β-CD and apoA-I on protein level of ABCA1 as the main protein involved in cholesterol efflux. Cultured astrocytes were treated with 5 µg/ml of apoA-I or 5 µM of β-CD for 24 hours. Following cell lysis, the lysates were loaded into SDS-PAGE and the protein level of ABCA1 was analysed by western blot. We found a significant increase in the ABCA1 protein (52%) after apoA-I treatment. However, β-CD significantly down regulated the protein level of ABCA1 compared with the control group (Fig .2).

### Cholesterol content in the cell and conditioned medium 

**Fig.3:**
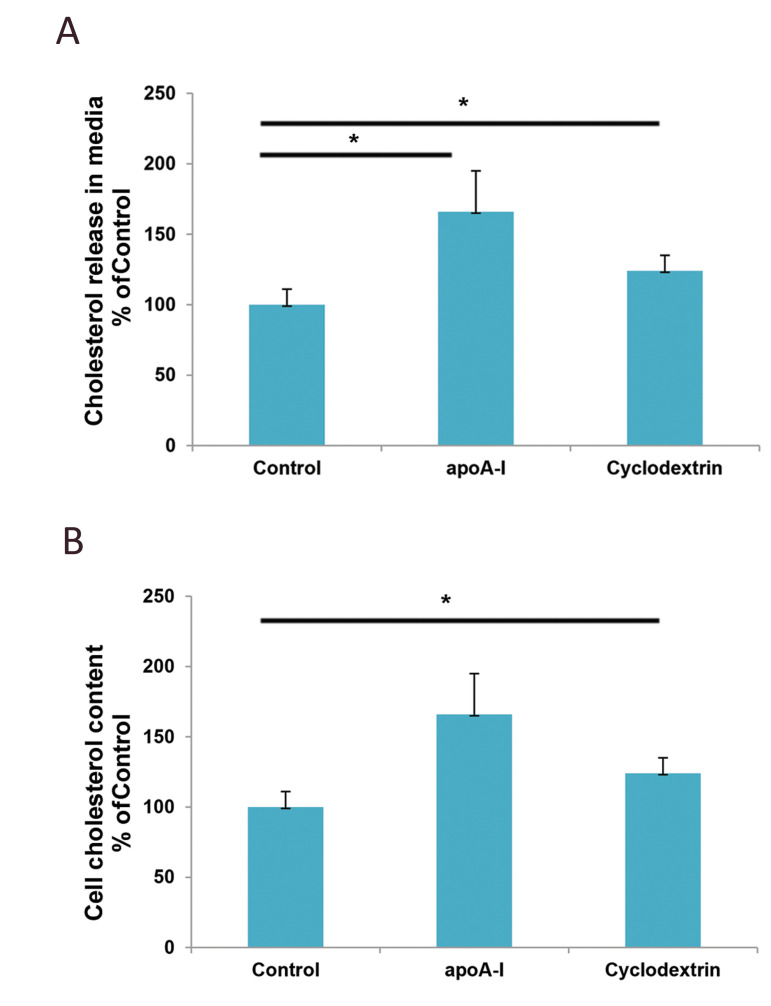
Effect of apoA-I and β-CD on the cell cholesterol content and cholesterol release in the media of astrocytes. Astrocyte-isolated newborn mice were incubated in the presence or absence of 5 μg/ml of apoA-I and 5 μM of β-CD. After 24 hours of incubation, **A. **we measured cholesterol release in the media and **B.** the cell cholesterol content according to the protocol in the Sigma cholesterol quantitation kit. Data were analysed with the student’s t-test and represent mean ± SD of triplicate samples. *P<0.05 and **P<0.01 indicate statistical significance. apoA-I; Apolipoprotein A-I, β-CD; Beta-cyclodextrin.

To determine the effect of apoA-I and β-CD on cholesterol release in conditioned medium and on cellular cholesterol content. a quantitative cholesterol kit (Sigma) was used following treatment with 5 µg/ml of apoA-I or 5 µM of β-CD for 24 hours. Cholesterol from both cells and media were extracted and further measured based on the protocol provided in the Sigma quantitative kit for the three experimental groups. Figure 3A shows a significant increase of approximately 66% in cholesterol level in the conditioned medium when the astrocytes were treated with apoA-I. β-CD increased cholesterol release to approximately 24%; however, it was still significant.

Our western blot data showed a significant increase in HMGCR after the astrocytes were treated with either apoA-I or β-CD. We checked to see if the HMGCR enhancement caused an abundance of cholesterol by assessing the cell cholesterol content in the treated astrocytes. Results shown in Figure 3B indicated an increase in cell cholesterol level by both apoA-I (about 15%) and β-CD (about 33%) in astrocytes compared with the control group. However, this increase was significant for β-CD, but not apoA-I (Fig .3B).

## Discussion

Abnormal accumulation of intracellular cholesterol results from impaired cholesterol trafficking/efflux (22). In healthy cells there are pathways involved in cholesterol delivery to the extracellular acceptors like apoA-I to provide a balance between cholesterol synthesis, trafficking, and efflux. This process regulates the cell cholesterol content and is mediated by many proteins, including HMGCR and ABCA1 as the two pivotal members of cholesterol homeostasis (7, 8). β-CD has been reported to be effective in regulating cholesterol metabolism (23), but relatively few studies have investigated its mechanism of action to influence *in vivo* or *in vitro* cholesterol metabolism, especially in the brain (24). The present study was carried out to investigate the effects of apoA-I, as a natural and well-established signal inducer for cell cholesterol homeostasis, and β-CD, as a cholesterol-lowering synthetic reagent, on protein levels of HMGCR and ABCA1 as a possible regulatory mechanism for cellular cholesterol depletion. 

Based on many reports, it is worth noting that apoA-I signalling activates the entire cholesterol metabolic cycle in astrocytes through promotion of cholesterol synthesis/ trafficking, and its subsequent efflux in order to inhibit cellular cholesterol accumulation. Here, we first checked the apoA-I signalling on protein level of ABCA1, HMGCR, and on cell cholesterol content and release. 

Our data showed that the ABCA1 protein level was significantly increased. There was a mild increase in HMGCR observed in astrocytes treated with apoA-I. Consistent with this finding, several studies have shown that apoA-I initially interacts with ABCA1 to generate HDL through promotion of cholesterol efflux (8). This interaction is believed to subsequently contribute to an increase in cellular content of ABCA1, suggesting the effect of apoA-I on stability of ABCA1 protein levels, which is in line with our results. HMGCR, along with cell cholesterol content and release were up regulated by apoA-I treatment, which suggested that the entire cell cholesterol pathway was under the control of apoA-I signalling in astrocytes. Astrocytes are the most abundant and supporting cells in the central nervous system (CNS). They should provide enough cholesterol to deliver cholesterol in the form of HDL cholesterol to the neurons (25). These results supported the findings of Ito et al. who reported increased synthesis of cholesterol and phospholipids in rat astrocytes after apoA-I treatment (26). 

β-CD, like apoA-I, is an acceptor for excess cell cholesterol (27); therefore, it is believed to be used as a cholesterol-lowering medicine in some neurodegenerative disease such as NPC to reduce cell overload cholesterol (19). Unlike the apoA-I effect, we observed an increased level of HMGCR and a decreased ABCA1 protein level in comparison to the control group in astrocytes treated with β-CD. In support of our findings, Coisne et al. reported a significant decrease of ABCA1 protein level in β-CDtreated bovine smooth muscle cells (24). Also, compared to apoA-I and in agreement with our western blot data, we observed a reduction in cholesterol release in conditioned media of astrocytes-treated with β-CD. This confirmed that ABCA1, which is the main protein responsible for cholesterol release, is affected by β-CD treatment. 

In contrast to the report showing that CD treatment blocked cholesterol efflux (28), our data demonstrated that CD, which is the cholesterol acceptor, significantly increased cholesterol secretion in conditioned media. β-CD could possibly deplete cholesterol just from plasma membrane because at the same time the cell cholesterol content is increased. Depletion of cholesterol from the plasma membrane may induce a positive feedback to increase HMGCR protein expression, and result in increased cholesterol synthesis. 

Overall, apoA-I regulates not only cholesterol efflux but also intracellular cholesterol trafficking and regulates all elements in cholesterol metabolism. However, due to the accumulation of cellular cholesterol, CD only releases cholesterol from the plasma membrane and does not support intracellular cholesterol trafficking. We have suggested that this regulation may be due to the decreased protein level of ABCA1 after CD treatment.

Since ABCA1 is involved in a variety of cell functions, its protein levels are tightly controlled by transcriptional and post-translational regulatory pathways (29). The cell cholesterol content in particular has a regulatory effect on ABCA1 abundance through the post-translational regulatory pathways. Although both apoA-I and β-CD are cholesterol acceptors that can deplete cell cholesterol (30) and increase cholesterol secretion in conditioned media, they have a different effect on ABCA1 abundance. Our findings suggest that, unlike apoA-I, β-CD lacks the ability to stabilize ABCA1, a crucial mediator of cholesterol efflux. Thus, it is likely that the action of β-CD inhibits ABCA1 signalling pathways, including cholesterol efflux, which results in abnormal cholesterol accumulation with long-term exposure. (31).

## Conclusion

Our study provides new evidence that β-CD, like apoA-I, can increase the HMGCR protein. Unlike apoA-I, it can reduce ABCA1, which may interfere with many cell functions and signalling that originate from ABCA1. Our findings are of great importance in the understanding of cellular events related to β-CD treatment. Further studies are necessary to clarify all unrecognized aspects of using CDs in treating neurodegenerative disorders like NPC and AD.

## Supplementary PDF


