# Supplementary material for: Impact of Methyl-β-Cyclodextrin and Apolipoprotein A-I on The Expression of ATP-Binding Cassette Transporter A1 and Cholesterol Depletion in C57BL/6 Mice Astrocytes
Source: Cell J. 2021 Mar 1;23(1):93–8. doi: 10.22074/cellj.2021.7061 (PMC7944131; doi:10.22074/cellj.2021.7061)
Supplement: Supplementary file 1 [file Cell-J-23-93-s01.pdf]

www.celljournal.org

Supplementary Information for

Impact of Methyl-β-Cyclodextrin and Apolipoprotein A-I on The Expression of ATP-Binding Cassette Transporter A1 and Cholesterol Depletion in C57BL/6 Mice Astrocytes

Shirin Azizidoost, M.Sc., Hossein Babaahmadi-Rezaei, Ph.D., Zahra Nazeri, M.Sc., Maryam Cheraghzadeh, Ph.D., Alireza Kheirollah, Ph.D.*

Department of Biochemistry, Cellular and Molecular Research Centre, Medical School, Ahvaz Jundishapur University of Medical Sciences, Ahvaz, Iran

**Corresponding Address: P.O. Box: 61357- 15794, Department of Biochemistry, Cellular and Molecular Research Centre, Medical School, Ahvaz Jundishapur University of Medical Sciences, Ahvaz, Iran*
Email: akheirollah@ajums.ac.ir

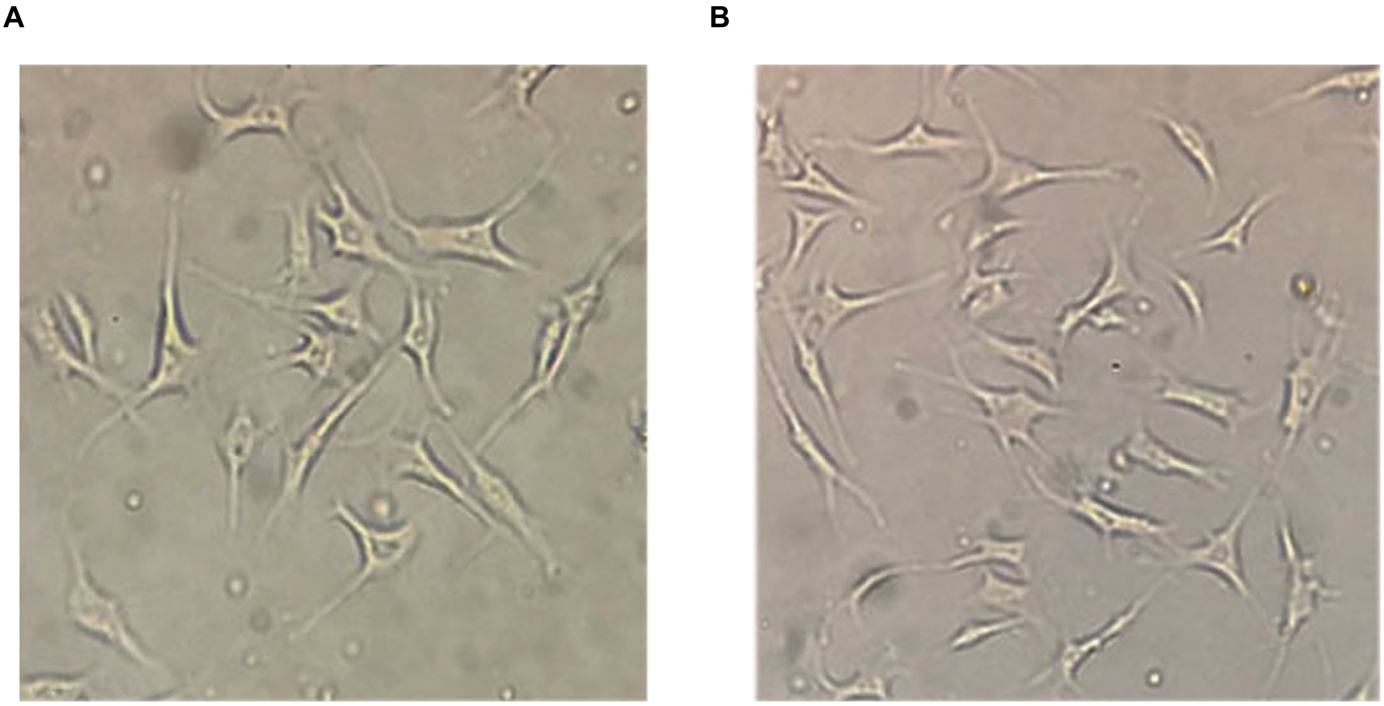

Fig.S1. Effect of treatment on astrocyte morphology. Light microscopy of astrocytes under 20x magnification. No morphology changes of astrocytes had been detected **A.** Before and **B.** After treatment (Scale bar=50 µm).
